# Prevalence of sarcopenia in community dwelling outpatient postmenopausal Hungarian women

**DOI:** 10.1186/s12891-022-05167-2

**Published:** 2022-03-04

**Authors:** Zoltán Pap, Irina Kalabiska, Ádám Balogh, Harjit Pal Bhattoa

**Affiliations:** 1 Kalman Laki Doctoral School of the University of Debrecen, Department of Traumatology and Hand Surgery, Faculty of Medicine, University of Debrecen, Debrecen, Hungary; 2 University of Physical Education, Research Center for Sport Physiology, Budapest, Hungary; 3 Regional Osteoporosis Center, Department of Obstetrics and Gynecology, Faculty of Medicine, University of Debrecen, Debrecen, Hungary; 4 Department of Laboratory Medicine, Faculty of Medicine, University of Debrecen, Nagyerdei Blvd. 98, Debrecen, H-4032 Hungary

**Keywords:** Sarcopenia, Appendicular skeletal muscle mass, Hand grip strength, Gait speed, Postmenopausal women

## Abstract

**Background:**

Ageing is an inherent feature of life and as per the United Nations, in the year 2020, 985 million women were ≥ 50 years of age worldwide, and the figure is expected to rise to 1.65 billion by 2050. Preservation of health and well-being in the elderly are challenging, and on the same note generalized changes in the musculoskeletal system contribute to this scenario. Musculoskeletal changes with ageing are referred to as sarcopenia. Reduced muscle mass and physical performance are hallmarks of sarcopenia, exclaimed with difficulty in independent activity and poor quality of life. Knowing that there is a hiatus in our knowledge as regards to the prevalence of sarcopenia in Hungary, the aim of this study was to determine the prevalence of sarcopenia in a community dwelling outpatient postmenopausal Hungarian cohort using the EWGSOP2 consensus recommendation.

**Methods:**

In this cross-sectional study, women arriving for routine bone densitometry examination at the Regional Osteoporosis Center, Department of Obstetrics and Gynecology, Faculty of Medicine, University of Debrecen were invited to participate in the study. A total of a 100 community-dwelling women were recruited who confirmed to the inclusion criteria of self-reported postmenopausal status, ≥ 50 years of age and gave written informed consent. The study procedures included the self-administered SARC-F questionnaire, followed by assessment of muscle strength, muscle quantity and physical preformance. Muscle strength was determined with the hand grip strength (HGS), appendicular skeletal muscle mass was assessed using dual energy X-ray absorptiometry and physical performance was determined by the gait speed (GS) test.

**Results:**

As per the EWGSOP2 definition, the percentage of study participants with probable sarcopenia (low muscle strength), sarcopenia (low muscle strength and low muscle quantity) and severe sarcopenia (low muscle strength, muscle quantity and low physical performance) was 36, 31 and 8%, respectively. Multiple linear regression analysis revealed that height, weight, HGS and GS were all independent predictors of appendicular skeletal muscle mass.

**Conclusion:**

The 31% prevalence of sarcopenia in the studied post-menopausal women highlights the need for adequate assessment of the condition in the elderly. Our findings most probably bear public health implications and may accelerate formulation of policies promoting healthy ageing.

**Supplementary Information:**

The online version contains supplementary material available at 10.1186/s12891-022-05167-2.

## Background

Rosenberg in his paper on the origins of the term sarcopenia cited the Greek words sarx and penia meaning flesh and loss, respectively; and the term reflects an age-related depletion of lean body mass inherently realized as alterations in mobility, nutritional intake and status, and over-all independence [1]. The definition of sarcopenia has since evolved with a dominant focus on muscle function, defined by muscle strength, muscle power or physical performance, as a potent predictor of relevant clinical outcomes rather than muscle mass alone [2–5]. Preservation of health and well-being in the elderly are challenging, and on the same note generalized changes in the musculoskeletal system contribute to this scenario. Depending on the definition used, the prevalence of sarcopenia ranges between 1 and 53% [6–42].

Sarcopenia is associated with a multitude of comorbidities having a significant impact on public health [43, 44]. The consequences of sarcopenia include high mortality, increased risk of falling, prolonged hospitalizations, augmented fracture risk, deprived mobility and physical function, and an inferior quality of life [6, 45–50].

As per an estimation by the United Nations, in the year 2020, 985 million women were ≥ 50 years of age worldwide, and the figure is expected to rise to 1.65 billion by 2050 [51]. As per the Hungarian 2011 consensus, 2,179,606 women were ≥ 50 years of age (21.9% of the population) [52]. This population may be considered postmenopausal as menopause is universal and shows meager variation in the timing of its onset, i.e., around 50 years of age, across human populations and has remained quite steady over the last 100 years in developed societies [53–55].

Studies suggest that the decline in estrogen levels during menopause may play a role in decline of lean body mass [56, 57]. Furthermore, postmenopausal women with sarcopenia were found to have decreased quality of life in a recent study by Cevei et al. [58].

In the past, diagnosis of sarcopenia was perhaps hindered by lack of a broadly accepted clinical definition. The European Working Group on Sarcopenia in Older People (EWGSOP) coined a definition that provided a framework for the diagnosis of sarcopenia in 2010 [59]. The definition was refined in 2019, and includes assessment of muscle strength, muscle mass and physical performance within a well-defined algorithm [60]. Although the EWGSOP2 consensus provides an exhaustive list of methodologies, they suggest hand grip strength determination for assessing muscle strength, dual-energy X-ray absorptiometry (DXA) to determine muscle quantity and gait speed to determine physical performance [60].

Knowing that there is a hiatus in our knowledge as regards to the prevalence of sarcopenia in Hungary, the aim of this study was to determine the prevalence of sarcopenia in a postmenopausal Hungarian cohort using the EWGSOP2 consensus recommendation.

## Methods

### Patients

In this cross-sectional study, women arriving for routine bone densitometry examination at the Regional Osteoporosis Center, Department of Obstetrics and Gynecology, Faculty of Medicine, University of Debrecen were invited to participate in the study. A total of a 100 community-dwelling women were recruited who confirmed to the inclusion criteria of self-reported postmenopausal status, ≥ 50 years of age and gave written informed consent. Self-reported menopause was defined as amenorrhea of at least 12 consecutive months after the last menstrual period [61]. Upon arrival to the out-patient department, patients were verbally informed about the study initiative with a brief summary on the importance of sarcopenia and a detailed description about the various study procedures. The study target of a 100 recruits was met in 3 months between January and March of 2019. None of the women declined invitation to participate in the study. The study was performed according to the Declaration of Helsinki and approved by the Ethics Committee of the University of Debrecen, Hungary (approval No. 5314–2019).

### Study procedures

The study commenced with the self-administered 5-item strength, assistance walking, rise from a chair, climb stairs and falls (SARC-F) questionnaire, followed by assessment of muscle strength, muscle quantity and physical preformance.

### SARC-F questionnaire

The SARC-F questionnaire is recommended by the EWGSOP2 as means of case-finding, where self-reports on signs that are characteristic of sarcopenia are noted from the patient [60]. The 5-item SARC-F questionnaire is a screening tool for risk of sarcopenia, where the patients insight of their extent in strength, walking ability, rising from a chair, stair climbing and acquaintances with falls is registered. As described by Malmstrom et al., the maximum SARC-F scale score is 10 (with 0–2 points for each of the 5 component; where a score of 0 is the best and 10 the worst) and a score of ≥4 is an indication for a diagnostic work-up to assess sarcopenia [62]. Strength is perceived by asking respondents how much difficulty they have lifting or carrying roughly 4.5 kgs i.e., 10 lbs. (0 = no difficulty, 1 = some, and 2 = a lot or unable to do). Assistance walking is assessed by noting the amount of difficulty the participant has when walking across a room and need for help or aids to do this (0 = no difficulty, 1 = some difficulty, and 2 = a lot of difficulty, use aids, or need of personal help). Rise from a chair pertains to the amount of difficulty in transferring from a chair or bed and whether they need help or aids to do this (0 = no difficulty, 1 = some difficulty, and 2 = a lot of difficulty, use aids, or need of help). Climb stairs is determined by querying the amount of difficultly the respondent has in climbing a flight of 10 steps (0 = no difficulty, 1 = some, and 2 = a lot or unable to do). For falls, reporting falling four times or more in the previous year is scored as 2, 1–3 times a year as 1, and for no reported falls as 0.

### Assessment of muscle strength

Muscle strength was determined with the hand grip strength (HGS) that was measured using the Jamar technologies hydraulic hand dynamometer (JLW Instruments, Chicago, IL, USA / Sammons Preston Rolyan, Bolingbrook, IL) as described by Roberts et al. [63]. In short, the patient is made comfortable in a chair with fixed legs and arms and a back support, with their forearms on the chair arms and their wrist, with their thumb facing upwards, over the end of the arms of the chair. The use of the dynamometer is demonstrated, and the importance of registering the best score upon tight gripping is emphasized. With the instrument comfortably in the patients’ right hand, the thumb is positioned around one side of the handle and the fingers around the other. In order to counteract the effect of gravity, the examiner rests the base of the instrument with his or her palm ensuring that its movement is not restricted. A strong squeeze is encouraged until the measuring needle stops rising. The grip strength is read from the outside dial in kilograms. The measurement is repeated for the left hand, with two additional measurements for each hand and the highest of the six readings and hand dominance is recorded.

### Assessment of the muscle mass

Dual-energy X-ray absorptiometry whole body scan was performed with the LUNAR Prodigy (GE-Lunar Corp., Madison, WI, USA) densitometer by a trained and certified DXA technician to assess the fat mass, lean mass, and bone mass of various regions of interest. Appendicular skeletal muscle mass (ASM) was calculated as the sum of lean mass in the arms and legs (all four extremities), assuming that all non-fat and non-bone tissue is skeletal muscle [64]. Since the EWGSOP2 criteria makes no recommendation for adjustment of body size, ASM (< 15 kg) per se was used to define sarcopenia [60].

### Assessment of physical performance

Physical performance was assessed using the 4 m usual walking speed test also known as the gait speed (GS) test [65, 66]. Patients covered 4 m on a straight clearly marked course in their usual pace, with a cane or walker if normally used, and the time taken was measured using a stopwatch. Speed was the distance travelled divided by the time taken measured by a stop watch.

As suggested by the EWGSOP2 consensus, the cut-off values for the measured variables are summarized in Table 1 [60].

**Table 1:** Indicator cut-off values for the studied parameters

Parameter	Cut-off values
Strength, assistance walking, rise from a chair, climb stairs and falls (SARC-F) questionnaire	≥4
Hand Grip Strength	< 16 kg
Appendicular skeletal muscle mass (ASM)	< 15 kg
4-m gait speed	≤0.8 m/s

As per the 2018 operational definition of sarcopenia, patients with low muscle strength are identified as those with probable sarcopenia, diagnosis of sarcopenia is considered confirmed by additional documentation of low muscle quantity or quality, additionally sarcopenia is considered severe when the patient presents with low muscle strength, muscle quantity or quality and low physical performance [60].

### Statistical methods

Descriptive statistics for all continuous variables are presented as median and range. The normality of distribution was assessed using the Kolmogorov-Smirnov test. The Spearman’s correlation coefficient was calculated for correlation analysis. Univariate and multiple regression analysis using the stepwise method was used to determine correlations and independent associations between parameters. Appendicular skeletal muscle mass was the dependent variables and SARC-F, weight, height, body mass index, HGS and GS were independent variables. The β standardized linear coefficients showing linear correlations between two parameters were determined. Independent association between the dependent and independent variables was indicated by the B (95%CI) regression coefficient. Statistical significance was defined as *p* values < 0.05. All analyses were performed using the SPSS Statistics software, version 25.0 (IBM Corps., Armonk, NY, USA).

## Results

**Fig. 1:**
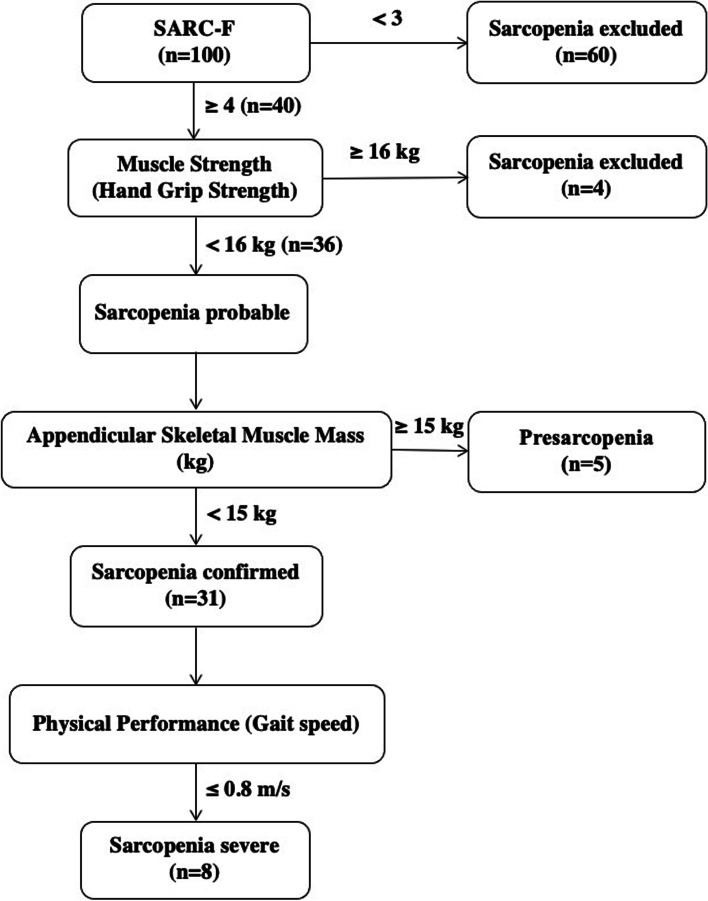
EWGSOP2 consensus recommended algorithm for sarcopenia case finding in the studied cohort

The characteristics of the patients in this cross-sectional study are shown in Table 2. As per the EWGSOP2 definition, the percentage of study participants with probable sarcopenia (low muscle strength), sarcopenia (low muscle strength and low muscle quantity) and severe sarcopenia (low muscle strength, muscle quantity and low physical performance) was 36, 31 and 8%, respectively (Fig. 1). On comparing study participants with low (< 15 kg) and normal (≥15 kg) ASM, significant differences were found in weight (57 (41–71) kg versus 67.5 (47–95) kg; *p* < 0.001), height (152.5 (141–170) cm versus 158 (146–169) cm; *p* < 0.001), body mass index (24.2 (19.2–31.3) kg/m^2^ versus 27.5 (18.8–36.5) kg/m^2^); *p* < 0.001), SARC-F questionnaire score (6 (4–9) versus 2 (0–7); *p* < 0.001), hand grip strength (12.6 (10.9–14.5) kg versus 21.2 (11.1–27.9) kg; *p* < 0.001) and gait Speed (1.05 (0.39–1.61) m/s versus 1.11 (0.9–1.61) m/s; *p* < 0.001). All parameters used in the calculation of ASM, i.e., left upper extremity lean mass, left lower extremity lean mass, right upper extremity lean mass and right lower extremity lean mass, were all statistically significantly lower in those with ASM < 15 kg. BMI adjusted ASM as suggested by Cawthon et al. was significantly lower in those with ASM < 15 kg [67].

**Table 2:** Patient Characteristics

Parameters (median, range)	All participants	ASM < 15 kg	ASM ≥15 kg
	(*n* = 100)	(*n* = 31)	(*n* = 69)
Age (years)	66 (50–84)	67 (50–83)	66 (50–84)
Weight (kg)	64.5 (41–95)	57 (41–71)^a^	68 (47–95)^a^
Height (cm)	157 (141–170)	152.5 (141–170)^b^	158 (146–169)^b^
Body mass index (kg/m^2^)	26.2 (18.8–36.5)	24.2 (19.2–31.3)^c^	27.5 (18.8–36.5)^c^
SARC-F questionnaire score	3 (0–9)	6 (4–9)^d^	2 (0–7)^d^
Hand grip strength (kg)	20.6 (10.9–27.9)	12.8 (10.9–14.5)^e^	21.2 (11.1–27.9)^e^
Gait Speed (m/s)	1.11 (0.39–1.61)	1.05 (0.39–1.61)^f^	1.11 (0.9–1.61)^f^
Left upper extremity lean mass (kg)	2.17 (1.29–3.09)	1.88 (1.29–2.28)^g^	2.30 (1.69–3.09)^g^
Left lower extremity lean mass (kg)	5.65 (3.50–9.33)	4.84 (3.50–5.44)^h^	5.97 (5.29–9.33)^h^
Right upper extremity lean mass (kg)	2.22 (1.49–3.17)	1.94 (1.55–2.30)^i^	2.36 (1.49–3.17)^i^
Right lower extremity lean mass (kg)	5.71 (3.70–9.54)	5.02 (3.70–5.61)^j^	6.01 (5.27–9.54)^j^
ASM (kg)	15.79 (10.81–24.10)	13.82 (10.81–14.88)^k^	16.41 (15.00–24.10)^k^
Body mass index adjusted ASM	0.610 (0.378–1.097)	0.577 (0.378–0.744)^l^	0.626 (0.435–1.097)^l^

^a,b,c,d,e,f,g,h,i,j,k,l^*p* < 0.001. ASM: Appendicular skeletal muscle mass. SARC-F: 5-item strength, assistance walking, rise from a chair, climb stairs and falls (SARC-F) questionnaire

Appendicular skeletal muscle mass showed statistically significant correlation with height, weight, body mass index, SARC-F questionnaire score, HGS and GS (Table 3).

**Table 3:** Correlation analysis between appendicular skeletal muscle mass and the dependent variables

		Height	Weight	BMI	SARC-F	HGS	GS
ASM	Spearman’s ρ *p* value	0.516 < 0.001	0.602 < 0.001	0.389 < 0.001	−0.612 < 0.001	0.628 < 0.001	0.347 < 0.001

*ASM* Appendicular skeletal muscle mass, *BMI* Body mass index, *SARC-F* 5-item strength, assistance walking, rise from a chair, climb stairs and falls (SARC-F) questionnaire, *HGS* Hand grip strength, *GS* Gait speed

Upon univariate analysis of the cohort data, participants with lower appendicular skeletal muscle mass had lower height, weight, body mass index, HGS and GS, and higher SARC-F score (Table 4).

**Table 4:** Results of the univariate analyses

Dependent variable	Independent variable	Univariate analysis		
		*B* (95% CI)	*β*	*p* value
Appendicular Skeletal Muscle Mass	Height	0.201 (0.143–0.259)	0.570	< 0.001
	Weight	0.121 (0.086–0.155)	0.575	< 0.001
	Body Mass Index	0.193 (0.083–0.303)	0.332	0.001
	SARC-F	−0.587 (− 0.742 - -0.432)	−0.605	< 0.001
	Hand Grip Strength	0.253 (0.187–0.319)	0.611	< 0.001
	Gait Speed	3.390 (1.676–5.103)	0.369	< 0.001

*SARC-F* 5-item strength, assistance walking, rise from a chair, climb stairs and falls (SARC-F) questionnaire

Multiple linear regression analysis revealed that height, weight, HGS and GS were independent predictors of appendicular skeletal muscle mass (Table 5).

**Table 5:** Multiple regression analysis of Appendicular Skeletal Muscle Mass

Dependent variable	Independent variable	Multivariate analysis		
		*B* (95% CI)	*β*	*p* value
Appendicular Skeletal Muscle Mass	Height	0.125 (0.073–0.176)	0.354	< 0.001
	Weight	0.046 (0.014–0.078)	0.219	0.006
	Hand Grip Strength	0.147 (0.087–0.207)	0.354	< 0.001
	Gait Speed	1.735 (0.479–2.990)	0.189	0.007

## Discussion

Using the algorithm detailed by the EWGSOP2, diagnostic work-up for sarcopenia was dictated in 40 (40%) study participants using the SARC-F questionnaire score, sarcopenia was probable in 36 of these individuals as per the muscle strength findings, sarcopenia was confirmed in 31 based on their muscle mass and 8 of them were diagnosed as having severe sarcopenia following assessment of their physical performance. Although the EWGSOP2 criteria do not explicitly define presarcopenia, those with a SARC-F score of ≥4 and ASM < 15 kg may well be categorized as presarcopenic, and are candidates for stringent follow-up in the near future. In our study, 5 individuals confirmed to the criteria of presarcopenia (Fig. 1).

The prevalence of sarcopenia has been reported by quite a few from various parts of the globe [6–58]. Table 6 lists the studies conducted in the past two decades. Table 6 summarizes the characteristics of the studied cohorts, the various techniques employed to assess muscle mass, muscle strength and physical performance, and the prevalence of sarcopenia for both sexes where available. Although the 2010 EWGSOP consensus delineates a frame-work for the assessment of sarcopenia, it is lenient in allowing the variables to be measured by various techniques that inherently leaves room for disparities as regards to comparison of published prevalence data [59]. The published data reports a prevalence of sarcopenia ranging between 1 and 53%, the aforementioned may offer explanation for this wide range, but other factors such as the variable age of the study participants and inclusion or non-inclusion of both sexes in the study cohort may also be contributory. It needs mention that apart from the EWGSOP consensus recommendations, the International working group on sarcopenia (IWG) and the Asian working group for sarcopenia (AWGS) recommendations are also in use [59, 68, 69]. The use of the International working group on sarcopenia criteria by Tramontano et al may not allow head-to-head comparison of the reported prevalence of sarcopenia in Peru to the other studies listed in Table 6. Furthermore, the Asian working group for sarcopenia recommendations have been used in quite a few Asian studies [40–42].

**Table 6:** Prevalence of Sarcopenia reported by studies in the last two decades

Study	Year	Country	Male/Female	Age, years mean (SD) [Range]	Assesment method			Sarcopenia prevalence, %		
					Muscle mass	Muscle strength	Physical performance	Total	Male	Female
Landi et al. [6]	2012	Italy	31/91	84.1 (4.8) [70+]	BIA	HGS	GS	32.8	67.7	20.8
Sanada et al. [7]	2012	Japan	0/533	[30–84]	DXA	HGS, LEP	Sit and reach, VO_2_ max	24.2	–	24.2
Tanimoto et al. [8]	2012	Japan	364/794	F: 73.9 (6.3) [65+]	BIA	HGS	GS	–	11.3	10.7
Abellan van Kan et al. [9]	2013	France	0/3025	80.51 (3.9) [≥75]	DXA	HGS	GS	5.2	–	5.2
Verschueren et al. [10]	2013	Belgium, UK	679/0	59.6 (10.7) [40–79]	DXA	HGS, IIS	GS	3.7	3.7	–
Patil et al. [11]	2013	Finland	0/409	74.2 (3.0) [70–80]	DXA	HGS	SPPB (4MGS, TUG, SB)	0.9–2.7	–	0.9
Landi et al. [12]	2013	Italy	118/236	85.8 (4.9)	MAMC	HGS	4MGS	29.1	27.1	19.8
Malmstrom et al. [13]	2013	USA	124/195	59.2 (4.4)	DXA	not included	4MGS, 6MWT	4.1	–	–
Legrand et al. [14]	2013	Belgium	103/185	84.8 (3.6) [> 80]	BIA	HGS	mSPPB, GS	12.5	14.6	12.4
Yamada et al. [15]	2013	Japan	568/1314	74.9 (5.5) [65–89]	BIA	HGS	10MGS	22	21.8	22.1
McIntosh et al. [16]	2013	Canada	42/43	75.2 (5.7)	BIA	HGS	DGI	6	5	7
Volpato et al. [17]	2014	Italy	250/288	77.1 (5.5) [65–89]	BIA	HGS	4MGS	10.2	7.6	12.5
Murphy et al. [18]	2014	USA	1426/1502	F: 73.8 (2.85)	DXA	HGS	GS	5	–	–
Jones et al. [19]	2015	UK	354/268	70.35 (9.2)	BIA	HGS	4MGS	14.5	11.8	17.4
Mijnarends et al. [20]	2016	Neatherlands	117/110	74.9 (7.2)	BIA	HGS	4MGS	23.3	23.9	22.7
Wang et al. [21]	2016	China	190/65	65.1 (10.85)	L3 TAMA	HGS	6MGS	12.5	13.7	9.2
Sousa et al. [22]	2016	Portugal	354/302	56 (22)	BIA	HGS	not included	24.2	28.5	19.2
Gani et al. [23]	2016	USA	608/561	62 [52–70]	L3 TPV	not included	not included	25.1	25	25.1
Wang et al. [24]	2016	China	462/482	69.02 (6.3) [60–92]	DXA, BIA	HGS	6MGS	10.4	8.2	12.5
Dorosty et al. [25]	2016	Iran	310/334	70.8 (6.1)	BIA	HGS	6MGS	16.5	21.3	8.9
Diz et al. [26]	2017	Brazil	2927/6459	83.4 (2.9)	DXA	not included	not included	17	12	20
Tramontano et al. [27]	2017	Peru	102/120	[65+]	BIA	not included	SPPB, 6MWT	17.6	0.01	31.7
Lou et al. [28]	2017	China	161/45	64 (10.1)	L3 TAMA	HGS	6MGS	6.8	5.6	11.1
Antunes et al. [29]	2017	Portugal	82/119	[65+]	MUAMC, MAMC	HGS	TUG	10.4	17.1	5.9
Kaplan et al. [30]	2017	USA	269/181	76 (68–89)	L3 TAMA	not included	not included	53.5	60.6	43.1
Huang et al. [31]	2017	China	364/106	65 (15)	L3 TAMA, VFA	HGS	6MWT	17	16.2	18.8
Lo et al. [32]	2017	Taiwan	689/648	[65+]	BIA	not included	not included	24.7	24.8	24.5
Dodds et al. [33]	2017	UK	282/437	[85+]	BIA	HGS	TUG converted to GS	20.7	20.9	20.6
Ngeuleu et al. [34]	2017	Marocco	16/107	52.3 (13.2)	DXA	not included	not included	39.8	56.3	37.4
Bianchi et al. [35]	2017	Italy	321/334	81 (6.8) [> 65]	BIA	HGS	4MGS	34.7	36.5	32.9
Martone et al. [36]	2017	Italy	183/211	79.6 (6.4)	BIA	HGS	4MGS	14.7	15.8	13.7
Bokshan et al. [37]	2017	USA	26/24	[55+]	L4 TPA	not included	not included	32	30.7	33.3
van Vugt et al. [38]	2018	Netherlands	149/75	[48–62]	L3 TAMA	not included	not included	24.6	24.8	24
Christensen et al. [39]	2018	Denmark	28/52	79 (6.6) [65–94]	DXA	HGS	10MGS	26.2	28.6	25
Chen et al. [40]	2018	China	228/148	64.33 (12.3)	TAMA, VFA	HGS	6MWT	24.5	19.3	32.4
Fung et al. [41]	2019	Singapur	206/181	68.3 (5.7) [60–89]	BIA	HGS	6MGS	27.4	21.8	33.7
Yang et al. [42]	2019	China	112/204	[60+]	BIA	HGS	4MGS	28.8	30.4	27.9
Our study		Hungary	0/100	67 [50–83]	DXA	HGS	GS	31	–	31

*BIA* Bioelectric impedance analysis, *HGS* Hand grip strength, *GS* Gait speed, *DXA* Dual energy X-ray absorptiometry, *LEP* Leg extension power, *VO*_*2*_ *max* Maximal oxygen uptake, *IIS* Isometric and isokinetic strength, *SPPB* Short physical performance battery, *4mGS* Gait speed (4 m), *TUG* Timed up and go test, *SB* Standing balance, *MAMC* Mid-arm muscle circumference, *6MWT* 6-min walk test, *mSPPB* Modified short physical performance battery, *10MGS* Gait speed (10 m), *DGI* Dynamic gait index, *L3 TAMA* Total abdominal muscle area at L3 lumbar spine level, *6MGS* Gait speed (6 m), *L3 TPV* Total psoas volume at L3 lumbar spine level, *MUAMC* Mid-upper-arm muscle circumference, *VFA* Visceral fat area, *L4 TPA* Total psoas area at L4 lumbar spine level

The probable limitations of our study are the relatively low number of participants, and the non-inclusion of men. The participants in this study were women who were referred for a routine bone densitometry examination and per se do not represent the general population. There may perhaps be a selection bias, since postmenopausal women reluctant to have routine checkups may have been missed from the study, moreover there may have been a greater likelihood of inclusion of those with musculoskeletal complaints. The reliability of self-reported menopausal status may be confounded by the subjective recollection of the reporter and perhaps complimentary assessment of folliculus stimulating hormone levels could have furthered more precise documentation of the the menopausal status of the studied women [70, 71]. The sample size of the studied population was limited to a 100 participants. The main aim of the present study was to estimate the prevalence of sarcopenia in a community dwelling postmenopausal out-patient population. For sample size calculation the expected proportion or prevalence in the population based to previous studies or pilot studies is a prerequisite [72]. There is no Hungarian data on the prevalence of sarcopenia, and knowing from other populations that previous estimates of prevalence of sarcopenia in women, from various age groups, range from 0.9–43.1% albeit based on different definitions of sarcopenia (Table 6), the sample size chosen in the present study would suffice to an estimated prevalence of below 7%. The finding of the present study at best qualify as findings from a pilot study given its inherent limitations and dictates validation on a sample size nurtured from the general population pool. Additional limitations include, lack of data on potential confounders such as total body fat, physical activity, diet and race.

Most studies report no significant association between sarcopenia prevalence and gender [8, 12, 14, 73, 74]. Nonetheless, Landi et al. reported that men were more commonly affected as compared to women [6] and Patel et al. in their study showed a higher prevalence of sarcopenia in women than in men [75]. This lack of consensus and the reported disparity among studies on the prevalence of sarcopenia in men and women advocate further investigations on the effect of gender on the prevalence of sarcopenia.

Estrogen decline, especially following menopause is one the factors implicated in the loss of lean muscle mass in women [76].

In our study, we used the EWGSOP2 recommendations published in 2019 [60]. As compared to the EWGSOP, the EWGSOP2 has better delineated the techniques recommended to assess muscle strength, muscle mass and physical performance [59, 60]. We used the DXA technique to assess muscle strength using the appendicular skeletal muscle mass. As recommended by the EWGSOP2 criteria, ASM in our cohort was not adjusted for body size [60]. Nonetheless, the use of unadjusted ASM to diagnose sarcopenia limits its comparability to previous studies where ASM was adjustment for height or body mass index.

Our findings most probably bear public health implications and may accelerate formulation of policies promoting healthy ageing.

## Conclusion

The 31% prevalence of sarcopenia in the studied post-menopausal women highlights the need for adequate assessment of the condition in the elderly.

## Supplementary Information

**Additional file 1.**

## Data Availability

All data generated or analyzed during this study are included in this published article and its supplementary information file.

## Abbreviations

10MGS: Gait Speed (10 m)
4MGS: Gait Speed (4 m)
6MGS: Gait Speed (6 Meters)
6MWT: 6-Minutes Walk Test
95%CI: 95 Percentage Confidence Interval
ASM: Appendicular Skeletal Muscle Mass
AWGS: Asian Working Group for Sarcopenia
BIA: Bioelectric Impedance Analysis
BMI: Body Mass Index
DGI: Dynamic Gait Index
DXA: Dual Energy X-Ray Absorptiometry
EWGSOP: European Working Group on Sarcopenia in Older People
EWGSOP2: European Working Group on Sarcopenia in Older People consensus of 2019
GS: Gait Speed
HGS: Hand Grip Strength
IIS: Isometric and Isokinetic Strength
IWG: International Working Group on Sarcopenia
L3 TAMA: Total Abdominal Muscle Area at L3 Lumbar Spine Level
L3 TPV: Total Psoas Volume at L3 Lumbar Spine Level
L4 TPA: Total Psoas Area at L4 Lumbar Spine Level
LEP: Leg Extension Power
MAMC: Mid-Arm Muscle Circumference
mSPPB: Modified Short Physical Performance Battery
MUAMC: Mid-Upper-Arm Muscle Circumference
SARC-F: 5-Item Strength, Assistance Walking, Rise from a Chair, Climb Stairs and Falls Questionnaire
SB: Standing Balance
SPPB: Short Physical Performance Battery
TUG: Timed Up and Go Test
VFA: Visceral Fat Area
VO_2_ max: Maximal Oxygen Uptake
WHO: World Health Organization
