# Supplementary material for: Prevalence of sarcopenia in community dwelling outpatient postmenopausal Hungarian women
Source: BMC Musculoskelet Disord. 2022 Mar 4;23:207. doi: 10.1186/s12891-022-05167-2 (PMC8897857; doi:10.1186/s12891-022-05167-2)
Supplement: Supplementary file 1 — Additional file 1. [file 12891_2022_5167_MOESM1_ESM.docx]

Supplementary data. All data generated or analyzed during the study.

| ID | DOE | Age | Weight | Height | BMI | Left_arm_Muscle_g | Left_leg_Muscle_g | Right_arm_Muscle_g | Right_leg_Muscle_g | ASM_kg | GS | HGS | SARC_F |
| --- | --- | --- | --- | --- | --- | --- | --- | --- | --- | --- | --- | --- | --- |
| 1 | 09.Jan.19 | 69 | 58 | 158 | 23.2 | 2070 | 5686 | 2114 | 5700 | 15.57 | 1.08 | 14.2 | 4 |
| 2 | 09.Jan.19 | 82 | 66 | 149 | 29.7 | 2250 | 5288 | 2451 | 5316 | 15.31 | 1.61 | 11.1 | 5 |
| 3 | 10.Jan.19 | 75 | 68 | 155 | 28.3 | 2414 | 6241 | 2660 | 6655 | 17.97 | 1.11 | 11.7 | 6 |
| 4 | 10.Jan.19 | 72 | 60 | 154 | 25.3 | 1973 | 4589 | 2064 | 4762 | 13.39 | 1.20 | 12.2 | 4 |
| 5 | 10.Jan.19 | 69 | 74 | 157 | 30.0 | 2458 | 5964 | 2564 | 6520 | 17.51 | 1.22 | 12.4 | 7 |
| 6 | 11.Jan.19 | 68 | 52 | 158 | 20.8 | 1882 | 4753 | 1898 | 4741 | 13.27 | 0.77 | 11.4 | 7 |
| 7 | 11.Jan.19 | 76 | 55 | 146 | 25.8 | 2005 | 5403 | 2191 | 4894 | 14.49 | 0.91 | 13.4 | 5 |
| 8 | 14.Jan.19 | 58 | 53 | 150 | 23.6 | 1885 | 4678 | 1817 | 4907 | 13.29 | 1.11 | 14.1 | 6 |
| 9 | 14.Jan.19 | 75 | 78 | 165 | 28.7 | 2668 | 6361 | 2964 | 6387 | 18.38 | 0.90 | 20.7 | 4 |
| 10 | 15.Jan.19 | 81 | 60 | 149 | 27.0 | 1872 | 4549 | 1918 | 5042 | 13.38 | 1.05 | 11.0 | 7 |
| 11 | 18.Jan.19 | 57 | 70 | 157 | 28.4 | 2156 | 5818 | 2342 | 5951 | 16.27 | 1.31 | 25.8 | 5 |
| 12 | 23.Jan.19 | 65 | 54 | 157 | 21.9 | 2227 | 5589 | 2228 | 5764 | 15.81 | 1.08 | 27.9 | 6 |
| 13 | 23.Jan.19 | 76 | 69 | 153 | 29.5 | 2086 | 5743 | 2393 | 5798 | 16.02 | 1.61 | 20.7 | 7 |
| 14 | 24.Jan.19 | 66 | 62 | 152 | 26.8 | 1945 | 5441 | 1977 | 5355 | 14.72 | 1.11 | 11.6 | 4 |
| 15 | 24.Jan.19 | 66 | 53 | 161 | 20.4 | 1712 | 4515 | 1844 | 4742 | 12.81 | 0.72 | 12.8 | 8 |
| 16 | 24.Jan.19 | 65 | 67 | 160 | 26.2 | 1980 | 4947 | 2237 | 5222 | 14.39 | 1.20 | 13.0 | 5 |
| 17 | 24.Jan.19 | 78 | 71 | 152 | 30.7 | 2181 | 4524 | 2123 | 4772 | 13.60 | 1.22 | 12.3 | 6 |
| 18 | 28.Jan.19 | 68 | 51 | 163 | 19.2 | 1826 | 5244 | 1906 | 5301 | 14.28 | 0.39 | 12.1 | 9 |
| 19 | 28.Jan.19 | 63 | 72 | 157 | 29.2 | 2618 | 5431 | 2309 | 5371 | 15.73 | 0.91 | 23.2 | 0 |
| 20 | 28.Jan.19 | 73 | 62 | 155 | 25.8 | 2260 | 5544 | 2379 | 5608 | 15.79 | 1.11 | 21.1 | 1 |
| 21 | 29.Jan.19 | 63 | 83 | 166 | 30.1 | 2835 | 6239 | 2682 | 6022 | 17.78 | 0.90 | 24.3 | 2 |
| 22 | 29.Jan.19 | 61 | 63 | 151 | 27.6 | 2100 | 5063 | 2062 | 5140 | 14.37 | 1.05 | 13.5 | 7 |
| 23 | 29.Jan.19 | 55 | 48 | 154 | 20.2 | 1796 | 4440 | 1914 | 4429 | 12.58 | 0.70 | 13.1 | 7 |
| 24 | 29.Jan.19 | 55 | 70 | 170 | 24.2 | 1817 | 5395 | 1943 | 5589 | 14.74 | 0.57 | 14.2 | 8 |
| 25 | 29.Jan.19 | 70 | 73 | 159 | 28.9 | 2426 | 7024 | 2334 | 7230 | 19.01 | 1.31 | 19.6 | 3 |
| 26 | 30.Jan.19 | 74 | 65 | 154 | 27.4 | 2173 | 4844 | 2299 | 4873 | 14.19 | 1.22 | 14.5 | 4 |
| 27 | 30.Jan.19 | 66 | 53 | 147 | 24.5 | 2013 | 4176 | 2297 | 4543 | 13.03 | 0.91 | 11.3 | 5 |
| 28 | 31.Jan.19 | 66 | 95 | 168 | 33.7 | 2573 | 6791 | 2604 | 7242 | 19.21 | 1.11 | 21.2 | 0 |
| 29 | 31.Jan.19 | 74 | 50 | 146 | 23.5 | 1513 | 3790 | 1801 | 3703 | 10.81 | 0.52 | 10.9 | 9 |
| 30 | 31.Jan.19 | 65 | 80 | 164 | 29.7 | 2948 | 6857 | 2384 | 6974 | 19.16 | 0.90 | 21.9 | 1 |
| 31 | 01.Feb.19 | 51 | 62 | 155 | 25.8 | 2470 | 5603 | 2456 | 5268 | 15.80 | 1.05 | 27.4 | 2 |
| 32 | 01.Feb.19 | 66 | 92 | 166 | 33.4 | 2638 | 6938 | 2542 | 7173 | 19.29 | 1.31 | 24.6 | 3 |
| 33 | 04.Feb.19 | 69 | 50 | 152 | 21.6 | 1476 | 4619 | 1761 | 5004 | 12.86 | 1.08 | 12.8 | 6 |
| 34 | 04.Feb.19 | 74 | 71 | 155 | 29.6 | 2185 | 5357 | 2473 | 5746 | 15.76 | 1.61 | 19.8 | 0 |
| 35 | 04.Feb.19 | 66 | 90 | 157 | 36.5 | 2484 | 5750 | 2176 | 5478 | 15.89 | 1.11 | 20.5 | 1 |
| 36 | 05.Feb.19 | 69 | 58 | 158 | 23.2 | 2075 | 5343 | 2220 | 5562 | 15.20 | 1.20 | 20.7 | 2 |
| 37 | 06.Feb.19 | 64 | 50 | 151 | 21.9 | 2570 | 5976 | 2919 | 6038 | 17.50 | 1.11 | 25.8 | 3 |
| 38 | 06.Feb.19 | 66 | 49 | 145 | 23.3 | 1644 | 3912 | 1621 | 3886 | 11.06 | 0.45 | 11.5 | 6 |
| 39 | 06.Feb.19 | 57 | 74 | 162 | 28.2 | 2261 | 6440 | 2884 | 6387 | 17.97 | 1.20 | 27.9 | 0 |
| 40 | 06.Feb.19 | 74 | 80 | 160 | 31.2 | 2000 | 6218 | 2273 | 5890 | 16.38 | 1.22 | 20.7 | 1 |
| 41 | 07.Feb.19 | 65 | 57 | 150 | 25.3 | 1996 | 6001 | 2056 | 5960 | 16.01 | 0.91 | 23.2 | 2 |
| 42 | 07.Feb.19 | 76 | 70 | 160 | 27.3 | 2273 | 5835 | 2293 | 5635 | 16.04 | 1.11 | 21.1 | 3 |
| 43 | 07.Feb.19 | 75 | 41 | 144 | 19.8 | 1493 | 4296 | 1547 | 4515 | 11.85 | 0.90 | 12.0 | 7 |
| 44 | 07.Feb.19 | 75 | 72 | 161 | 27.8 | 3090 | 9334 | 2130 | 9545 | 24.10 | 1.05 | 24.3 | 1 |
| 45 | 08.Feb.19 | 57 | 49 | 153 | 20.9 | 2609 | 6017 | 2615 | 5659 | 16.90 | 1.31 | 19.6 | 2 |
| 46 | 08.Feb.19 | 64 | 67 | 158 | 26.8 | 2293 | 5431 | 2285 | 5952 | 15.96 | 1.08 | 21.2 | 3 |
| 47 | 13.Feb.19 | 65 | 69 | 151 | 30.3 | 2529 | 6676 | 2683 | 6502 | 18.39 | 1.61 | 21.9 | 1 |
| 48 | 13.Feb.19 | 55 | 47 | 151 | 20.6 | 1946 | 5174 | 1865 | 5082 | 14.07 | 1.61 | 13.0 | 4 |
| 49 | 14.Feb.19 | 57 | 51 | 156 | 21.0 | 2061 | 5716 | 2242 | 5636 | 15.66 | 1.11 | 24.6 | 2 |
| 50 | 14.Feb.19 | 66 | 44 | 146 | 20.6 | 1694 | 4384 | 2038 | 4775 | 12.89 | 1.20 | 14.1 | 5 |
| 51 | 14.Feb.19 | 66 | 59 | 159 | 23.3 | 2033 | 5242 | 2068 | 5394 | 14.74 | 1.22 | 11.0 | 6 |
| 52 | 18.Feb.19 | 72 | 64 | 143 | 31.3 | 1767 | 4763 | 1914 | 5036 | 13.48 | 0.91 | 11.4 | 7 |
| 53 | 18.Feb.19 | 76 | 70 | 151 | 30.7 | 2418 | 5330 | 2181 | 5432 | 15.36 | 1.11 | 19.8 | 3 |
| 54 | 19.Feb.19 | 76 | 50 | 153 | 21.4 | 2176 | 5080 | 2107 | 5017 | 14.38 | 0.90 | 12.8 | 4 |
| 55 | 19.Feb.19 | 63 | 47 | 158 | 18.8 | 2932 | 7323 | 2585 | 7791 | 20.63 | 1.05 | 20.5 | 1 |
| 56 | 19.Feb.19 | 60 | 55 | 152 | 23.8 | 2278 | 5330 | 2012 | 5209 | 14.83 | 1.31 | 12.1 | 5 |
| 57 | 19.Feb.19 | 62 | 53 | 159 | 21.0 | 2189 | 5313 | 2211 | 5563 | 15.28 | 1.22 | 20.7 | 2 |
| 58 | 19.Feb.19 | 69 | 63 | 159 | 24.9 | 2142 | 5711 | 2342 | 5692 | 15.89 | 1.31 | 25.8 | 3 |
| 59 | 19.Feb.19 | 70 | 80 | 150 | 35.6 | 2169 | 5825 | 2043 | 6168 | 16.21 | 1.08 | 27.9 | 1 |
| 60 | 20.Feb.19 | 63 | 66 | 165 | 24.2 | 2449 | 6216 | 2737 | 6839 | 18.24 | 1.61 | 20.7 | 2 |
| 61 | 20.Feb.19 | 68 | 60 | 162 | 22.9 | 2171 | 6104 | 1920 | 6219 | 16.41 | 1.11 | 23.2 | 3 |
| 62 | 21.Feb.19 | 64 | 71 | 165 | 26.1 | 2302 | 5945 | 1927 | 6177 | 16.35 | 1.20 | 21.1 | 1 |
| 63 | 21.Feb.19 | 65 | 60 | 156 | 24.7 | 2185 | 5565 | 1919 | 5430 | 15.10 | 1.11 | 24.3 | 2 |
| 64 | 21.Feb.19 | 64 | 64 | 169 | 22.4 | 2687 | 6742 | 2488 | 6624 | 18.54 | 1.20 | 19.6 | 3 |
| 65 | 21.Feb.19 | 70 | 57 | 156 | 23.4 | 2755 | 6304 | 2780 | 6525 | 18.36 | 1.22 | 21.2 | 1 |
| 66 | 21.Feb.19 | 55 | 77 | 162 | 29.3 | 2493 | 7069 | 2439 | 7270 | 19.27 | 0.91 | 21.9 | 2 |
| 67 | 21.Feb.19 | 67 | 59 | 158 | 23.6 | 2510 | 6069 | 2734 | 5994 | 17.31 | 1.11 | 27.4 | 3 |
| 68 | 22.Feb.19 | 75 | 66 | 157 | 26.8 | 2559 | 6763 | 2989 | 6978 | 19.29 | 1.05 | 24.6 | 1 |
| 69 | 25.Feb.19 | 63 | 75 | 150 | 33.3 | 2429 | 5728 | 2467 | 6010 | 16.63 | 1.31 | 19.8 | 2 |
| 70 | 25.Feb.19 | 63 | 74 | 167 | 26.5 | 2298 | 6090 | 2546 | 6410 | 17.34 | 1.08 | 20.5 | 3 |
| 71 | 25.Feb.19 | 50 | 64 | 168 | 22.7 | 2583 | 6123 | 2322 | 6290 | 17.32 | 1.61 | 20.7 | 2 |
| 72 | 27.Feb.19 | 69 | 80 | 161 | 30.9 | 2651 | 7297 | 2537 | 7511 | 20.00 | 1.61 | 25.8 | 3 |
| 73 | 27.Feb.19 | 70 | 65 | 153 | 27.8 | 2304 | 5473 | 2208 | 5595 | 15.58 | 1.11 | 27.4 | 2 |
| 74 | 01.Mar.19 | 64 | 78 | 163 | 29.4 | 2634 | 6412 | 2528 | 6735 | 18.31 | 1.20 | 24.6 | 3 |
| 75 | 04.Mar.19 | 67 | 73 | 161 | 28.2 | 2174 | 5776 | 2698 | 6357 | 17.01 | 1.22 | 19.8 | 2 |
| 76 | 04.Mar.19 | 67 | 63 | 158 | 25.2 | 1726 | 5157 | 1932 | 5394 | 14.21 | 0.91 | 13.0 | 6 |
| 77 | 04.Mar.19 | 72 | 56 | 156 | 23.0 | 2143 | 5713 | 2152 | 5961 | 15.97 | 1.11 | 20.5 | 3 |
| 78 | 04.Mar.19 | 70 | 73 | 162 | 27.8 | 2177 | 6291 | 2195 | 6384 | 17.05 | 1.31 | 20.7 | 1 |
| 79 | 04.Mar.19 | 68 | 60 | 162 | 22.9 | 2045 | 5904 | 1489 | 5608 | 15.05 | 1.08 | 25.8 | 2 |
| 80 | 05.Mar.19 | 83 | 66 | 155 | 27.5 | 2036 | 6132 | 2316 | 6035 | 16.52 | 1.61 | 27.9 | 1 |
| 81 | 05.Mar.19 | 60 | 57 | 153 | 24.3 | 1704 | 5114 | 1802 | 5449 | 14.07 | 1.11 | 13.2 | 7 |
| 82 | 05.Mar.19 | 62 | 86 | 166 | 31.2 | 3048 | 7514 | 2584 | 7780 | 20.93 | 1.20 | 20.7 | 2 |
| 83 | 05.Mar.19 | 65 | 66 | 155 | 27.5 | 2182 | 5419 | 3167 | 5511 | 16.28 | 1.11 | 23.2 | 1 |
| 84 | 06.Mar.19 | 59 | 62 | 169 | 21.7 | 2221 | 7381 | 2436 | 7675 | 19.71 | 1.20 | 21.1 | 2 |
| 85 | 07.Mar.19 | 59 | 65 | 156 | 26.7 | 2168 | 5766 | 1895 | 6165 | 15.99 | 1.22 | 24.3 | 1 |
| 86 | 07.Mar.19 | 65 | 70 | 157 | 28.4 | 2171 | 5353 | 2411 | 5484 | 15.42 | 0.91 | 19.6 | 2 |
| 87 | 08.Mar.19 | 61 | 74 | 151 | 32.5 | 2129 | 5621 | 2165 | 5585 | 15.50 | 1.11 | 21.2 | 1 |
| 88 | 08.Mar.19 | 53 | 57 | 161 | 22.0 | 1752 | 5083 | 1747 | 5243 | 13.83 | 0.49 | 12.9 | 7 |
| 89 | 08.Mar.19 | 71 | 67 | 167 | 24.0 | 2334 | 5398 | 2223 | 5722 | 15.68 | 1.11 | 21.9 | 2 |
| 90 | 08.Mar.19 | 58 | 59 | 146 | 27.7 | 2152 | 5364 | 2509 | 5728 | 15.75 | 1.20 | 24.6 | 1 |
| 91 | 08.Mar.19 | 82 | 58 | 141 | 29.2 | 1290 | 3504 | 2043 | 4201 | 11.04 | 1.11 | 14.2 | 4 |
| 92 | 11.Mar.19 | 77 | 64 | 159 | 25.3 | 2052 | 5216 | 1973 | 5371 | 14.61 | 1.20 | 11.1 | 5 |
| 93 | 11.Mar.19 | 73 | 72 | 157 | 29.2 | 2307 | 6074 | 2544 | 5980 | 16.91 | 1.22 | 19.8 | 2 |
| 94 | 11.Mar.19 | 64 | 69 | 162 | 26.3 | 1695 | 5409 | 2122 | 5773 | 15.00 | 0.91 | 11.7 | 6 |
| 95 | 11.Mar.19 | 63 | 64 | 165 | 23.5 | 2144 | 5753 | 2005 | 5784 | 15.69 | 1.11 | 20.5 | 1 |
| 96 | 11.Mar.19 | 73 | 70 | 164 | 26.0 | 2166 | 6453 | 2276 | 6730 | 17.63 | 1.05 | 20.7 | 2 |
| 97 | 12.Mar.19 | 72 | 67 | 157 | 27.2 | 2367 | 6186 | 2360 | 6234 | 17.15 | 1.31 | 25.8 | 2 |
| 98 | 12.Mar.19 | 50 | 62 | 159 | 24.5 | 1931 | 5304 | 2035 | 5608 | 14.88 | 1.08 | 12.4 | 7 |
| 99 | 12.Mar.19 | 74 | 80 | 152 | 34.6 | 2431 | 6353 | 2192 | 6163 | 17.14 | 1.22 | 27.9 | 3 |
| 100 | 12.Mar.19 | 64 | 67 | 160 | 26.2 | 2101 | 5596 | 2029 | 5960 | 15.69 | 0.91 | 20.7 | 2 |
